# Biomedical Waste Management: A Study of Knowledge, Attitude, and Practices in a Tertiary Health Care Institution in Bijapur

**DOI:** 10.4103/0970-0218.62591

**Published:** 2010-01

**Authors:** MC Yadavannavar, Aditya S Berad, PB Jagirdar

**Affiliations:** Department of Community Medicine, Shri B M Patil Medical College, Bijapur, Karnataka, India

## Introduction

Health care waste refers to all the waste generated by a health care establishment. It is estimated that 10-25% of health care waste is hazardous, with the potential for creating a variety of health problems.(1) Bio-medical waste (BMW) collection and proper disposal has become a significant concern for both the medical and the general community.(2) Since the implementation of the Biomedical Waste Management and Handling Rules (1998),(3) every concerned health personnel is expected to have proper knowledge, practice, and capacity to guide others for waste collection and management, and proper handling techniques. Bijapur district in the northern part of Karnataka State has two district hospital and Medical college hospital, including a secondary level health care service. This study was undertaken to assess the knowledge, attitude, and practices (KAP) of the employees of Bijapur liberal district education association (BLDEA's) Shri BM Patil Medical College and Hospital, Bijapur. This cross-sectional study was carried out in the month of November 2007. A total of 334 employees were surveyed, out of which 180 were non-teaching and 154 were teaching staff. A pretested, self-administered questionnaire containing questions on KAP regarding bio-medical waste management was used. Before administering the questionnaire the purpose of the study was explained to all participating employees. For statistical analysis we calculated percentages and applied the Chi-square test. Anonymity of the participants was maintained throughout the study.

## Results

The teaching staff of the hospital gave more correct responses (97.4%) to questions on BMW management than the nonteaching staff (80%). This difference was statistically significant (*P* < 0.01).

Similar differences were observed between teaching and non-teaching staff with respect to attitude and practices regarding BMW management (*P* < 0.01).

## Discussion and Conclusion

The participants involved in this study were assessed knowledge, attitude, and practice of BMW management. Interestingly, this study revealed that the awareness and proper practice of BMW was very satisfactory. Our study showed that the majority of staff (teaching and non-teaching) were conscious of the measures for safe collection and final disposal of BMW; this is in contrast to the finding reported by Pandit *et al*.(4) Since the Shri BM Patil Medical College and Hospital has its own incineration plant, all the employees of various designations are required to ensure proper collection, segregation, and transport to the final disposal point. Panditet*et al*.(4) reported that proper hospital waste management was not being practiced. It is possible that the newer employees are being oriented regarding proper collection and transport of hospital waste. Findings similar to that in our study were observed by Rao.(5) In this study a need to periodically acquaint the participants with the updated BMW management and handling rules was felt.

We recommend that strict supervision and surveillance be followed in day-to-day hospital waste management activities.
